# Interposition of Ileal J-Pouch for Rectum Reconstruction in Dog

**Published:** 2014-03

**Authors:** Leila Ghahramani, Saeed Yazdani, Saeed Derakhshani, Abbas Rezaianzadeh, Reza Jalli, Bita Geramizadeh, Ali Reza Safarpour, Salar Rahimikazerooni, Seyed Vahid Hosseini

**Affiliations:** 1 Colorectal Research Center, Faghihi Hospital, Shiraz University of Medical Sciences, Shiraz, Iran;; 2 Department of General Surgery, Chamran Hospital, Tehran, Iran;; 3 Research Center for Health Sciences, School of Health, Shiraz University of Medical Sciences, Shiraz, Iran;; 4 Department of Radiology, Nemazee Hospital, Shiraz University of Medical Sciences, Shiraz, Iran;; 5 Department of Pathology, School of Medicine, Shiraz University of Medical Sciences, Shiraz, Iran

**Keywords:** Rectum, Ileal pouch, Reconstruction

## Abstract

**Background:** The gold standard of the management of rectal cancer in the middle and lower parts is low anterior resection with coloanal anastomosis. About 50% of the patients undergoing this procedure might experience some complications because of the low capacity of the neorectum. The aim of this study was to evaluate ileal J-pouch interposition as a neorectum between the anal canal and the remaining colon in comparison to coloanal anastomosis and transverse coloplasty.

**Methods: **Twelve dogs, weighing 23-27 kg, were divided into three groups. After laparotomy, the volume of the primary rectum was measured so that it could be compared with that of the neorectum at the end of the study. After rectal resection in Group A, the colon was directly anastomosed to the anus. In Group B, a 5-cm longitudinal incision was made 2 cm proximal to the anastomosis and was sutured transversely (coloplasty). In Group C, a 5-cm ileal J-pouch was interposed between the colon and anus. After 8 weeks, the neorectum was evaluated for volume, radiology, and pathology.

**Results: **All the samples were alive until the end of the study. The healing of the anastomotic lines was acceptable (pathologically) in all. The mean volume expansion was 20.9% in Group A, 21.7% in Group B, and 118.2% in Group C, with the latter being significantly higher than that of the other groups (P=0.03). Colon J-pouch and coloplasty after proctectomy in some situations have not been performable. This study evaluated the performance of ileal J-pouch interposition.

**Conclusion: **This study showed that ileal J-pouch interposition might produce an acceptable reservoir function and that it seems feasible and safe in selected cases.

## Introduction

The gold standard of low rectal cancer has been low anterior resection with total mesorectal excision. The neorectum is constructed with the remaining left colon. However, almost all of the patients with straight coloanal anastomosis have tolerated some complications of defecation after low anterior resection such as urgency, frequency, soiling, anastomotic line stenosis because of the vascular tension of the sigmoid colon, and incontinence due to the low capacity of the neorectum.^1^^,^^2^ Some procedures have been described to resolve this low compliance, including side-to-end coloanal anastomosis, transverse coloplasty, and colonic J-pouch.^3^ Small colonic J-pouch has had an optimal function in that it can reduce difficult evacuation from 30% to 10% at long-term follow-up.^4^^,^^5^ Nevertheless, in some situations, similar to previous colonic resection, owing to diffuse diverticulosis, bulky sphincter, insufficient colon length, pregnancy, complex surgery and narrow pelvis, surgeons cannot reconstruct the reservoir with the colon.^3^

Accordingly, in this animal pilot study, we tried to construct the neorectum with the interposition of the ileal J-pouch between the anal canal and the remaining colon. We thereafter evaluated the efficacy of the ileal J-pooch as a neorectum in comparison to straight coloanal anastomosis and transverse coloplasty. 

## Materials and Methods

*Materials*

The present study was performed in the Animal House of Shiraz University of Medical Sciences (South of Iran) in August, September, and October 2011 and was approved by the Ethics Committee of Shiraz University of Medical Sciences for the use of laboratory animals.   

In this study, 12 German shepherd dogs, weighing 23-27 kg, were selected and divided into three equal groups. 

*Surgical Procedure*

All the dogs were anesthetized with 17 mg/kg of thiopental intravenously. After intubation, anesthesia was continued through a mixture of halothane and oxygen up to the end of the operation. Hydration of the samples was maintained by 1000 cc of D/S during the operation. Additionally, half of a pen-and-strep vial (3 million units of penicillin plus 3 g of streptomycin) and a 1 g of Keflin were injected preoperatively. Then via a midline laparotomy incision, a clamp was placed 10 cm proximal to the anus. An orotracheal tube (OT tube) was entered into the rectum, and 10 ml of air was injected into its cuff. In order to prevent leakage, a purse-string suture was placed around the anus with nylon 1. Moreover, the volume of the primary rectum was measured to be compared with that of the neorectum at the end of the study. To measure the volume of the rectum, N/S solution was injected into the rectum in the form of free fluid through a bottle, which was placed 100 cm above the anus level. After fullness of the rectum, the volume of the primary rectum was measured and recorded. In fact, the volume of the primary rectum was equal to the volume of the injected N/S plus the 10 ml of air injected into the OT tube. Afterwards, the rectum was emptied, the OT tube was also removed, and the intended operations were performed in each group.^1^ In all the cases, the rectum was resected 2 centimeters above the dentate line. The proximal margin was resected at the level of the sacral promontory. Total mesorectal excision was done.^1^^,^^6^ After the resection of the rectum, in Group A the colon was directly anastomosed to the anus. In Group B, however, a 5-cm longitudinal incision was made 2 cm proximal to the anastomosis and was transversely sutured (coloplasty).^3^ In Group C, 10 centimeters of the terminal ileum with the main branch of the ileocolic vessel was separated.^7^ After the creation of a J-pouch by the terminal ileum, the pouch was applied in the pelvis and anastomosis was done with Prolyn 3/0.

*Postoperative Management*

After irrigation and hemostasis, the abdominal wall was closed in layers and tetracycline was sprayed on the wound. The animals were kept NPO and were given intravenous fluid (D/S) for 3 days with half of a pen-and-strep vial intramuscularly per day for 10 days postoperatively. After 3 days, a soft food diet without bones was started for them for 7 days. Then, they were given normal food up to the end of the study. The samples were kept in the same condition for 8 weeks. Afterwards, the volume of the neorectum was measured and recorded. Furthermore, the neorectum was removed for pathological and radiographic evaluations.

*Statistical Analysis*

A non-parametric Mann-Whitney U test with SPSS (version 18) was used for data analysis. Significance level was considered as 0.05. 

## Results

All the dogs were alive until the end of the study with a good condition. According to the pathological reports, the healing of the proximal anastomosis was acceptable in all the three groups under study. tables 1 and 2 show the basis for pathological grading.

**Table 1:** Pathological characteristics of the samples

**Group**	**Subgroup**	**Pathology finding**	**Pathology grading**
A	A1	Granulation tissue, mild inflammation	II
	A2	Complete healing process (fibrosis)	I
	A3	Mild to moderate inflammation, loose GT	III
	A4	Granulation tissue, no inflammation	I-II
B	B1	Moderate inflammation, granulation tissue	III
	B2	Mild inflammation, granulation tissue	II
	B3	Moderate inflammation, no granulation	III
	B4	Moderate inflammation, loose GT	III
C	C1	Moderate inflammation, loose granulation tissue	III
	C2	Severe inflammation, ulceration and fibrin exudate	IV
	C3	Mild to moderate inflammation, no granulation tissue	II-III
	C4	Ulceration + fibrin exudate and focal necrosis	IV

**Table 2:** Basis for pathological grading

**Grade**	**Characteristics **
I	Complete healing, fibrosis, or epithelialization
II	Mild inflammation, granulation tissue
III	Moderate inflammation, loose granulation tissue
IV	Sever inflammation, necrosis, ulceration, no granulation tissue

**Figure 1:**
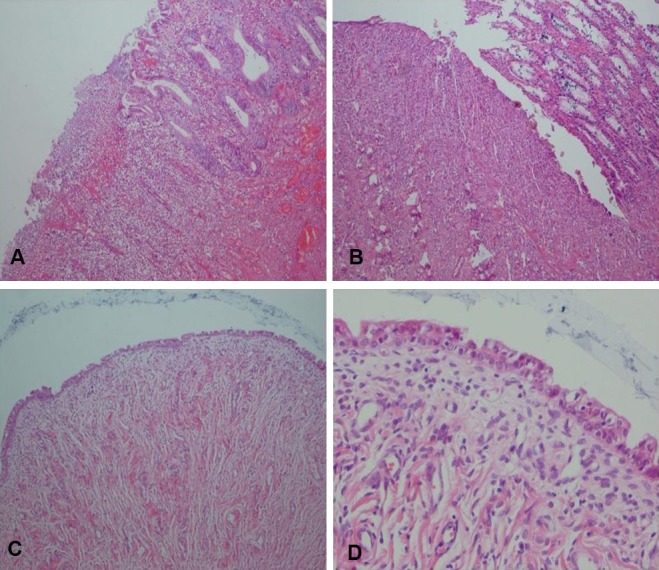
Ulceration, congestion, and inflammation in Group C (H&E x100) (A). Repair with connective tissue in Group C: anastomotic line (H&E x100) (B). Repair and surface epithelialization in Group C (small bowel epithelium) (H&E x100) (C). Epithelialization plus dense granulation tissue (repair) (H&E x400) (D).

**Figure 2:**
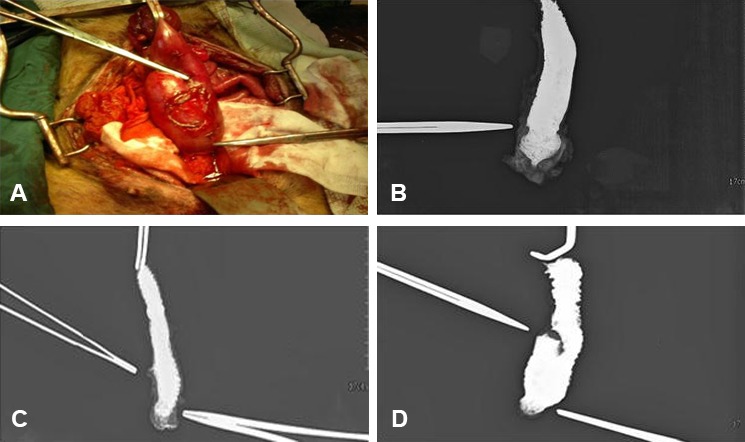
Visible intact pouch before excision (A). Contrast study of the direct coloanal anastomosis specimen (B). Contrast study of the coloplasty specimen (C). Contrast study of the ileal J pouch specimen (D).

Inflammation and ulceration was detected in the samples taken from the pouch (figure 1A). Repair of the connective tissue was also seen at the site of anastomosis (figure 1B) and on the mucosal surface (figure 1C) in the suture line of the pouch. Deep biopsy of the pouch showed that repair included both epithelialization and dense fibroblast tissue (figure 1D). The pouch which was made for Group C was completely intact with a proper vascularization (figure 2A).

After biopsy, all the samples were filled by contrast and evaluated by an expert radiologist. In comparison, the volume increase in the pouch group (figure 2B) was markedly higher than the volume increase in the coloplasty (figure 2C) and direct anastomosis Groups (figure 2D).

The dogs’ weights in the three groups under study were not markedly different. The primary volume of the rectum, volume after 8 weeks (end of the study), and volume increase for each dog were measured. The volume increase in each group was also calculated (table 3).

**Table 3:** Volume of the primary rectum and neorectum in all the three groups under study

**Number**	**Weight (kg)**	**Volume of the primary rectum (cc)**	**Rectum volume after 8 weeks (end of the study)**	**Volume increase**	**Mean volume ** **increase within groups**
A1	23	150	180	20%	20.98%
A2	26	150	200	33.3%	20.98%
A3	23	140	150	7.1%	20.98%
A4	27	170	210	23.5%	20.98%
B1	27	160	180	12.5%	21.75%
B2	23	130	150	15.3%	21.75%
B3	24	140	180	28.5%	21.75%
B4	23	130	170	30.7%	21.75%
C1	24	170	350	105.8%	118.27%
C2	27	155	380	145.1%	118.27%
C3	27	150	300	100%	118.27%
C4	23	90	200	122.2%	118.27%

Considering Group A (the control group), the percentage of the increase in the volume of the rectum (the volume of the primary rectum in comparison to the volume of the neorectum at the end of the study) was as follows:

A1: 150cc            180cc (20% ↑)

A2: 150cc             200cc (33% ↑)

A3:140cc              150cc (7.1% ↑)

A4: 170cc             210cc (23.5% ↑)

Moreover, the mean volume increase in Group A was measured as 20.9%.

The percentage of the volume increase in the place of the rectum in Group B (the coloplasty group) was as follows:

B1: 160cc           180cc (12.5% ↑)

B2: 130cc          150cc (15% ↑)

B3: 140cc          180cc (28.5% ↑)

B4: 130cc         170cc (31% ↑)

In addition, the mean volume increase in Group B was equal to 21.7%.

Finally, the percentage of the volume increase in the place of the rectum in Group C (J-pouch) was as follows:

C1:170cc           350cc (106% ↑)

C2:155cc           380cc (145% ↑)

C3:150cc           300cc (100% ↑)

C4: 90cc            200cc (122% ↑)

Also, the mean volume increase in Group C was 118.2%.

## Discussion

Although colon J-pouch is the best method of operation after removing the rectum, J-pouch coloanal anastomosis was not possible in 26.2% of low rectal cancer patients who had undergone low ant resection plus total mesorectal excision.^3^ This situation occurs in the following conditions:

Narrow pelvic, Bulky sphincter, Diverticulitis, Insufficient colon length, Pregnancy, Complex surgery, Distant metastasis^3^

Nowadays, the low ant resection operation, accompanied by total mesorectal excision (TME) is considered the standard treatment for rectal cancers. Moreover, in order to decrease the complications of the direct anastomosis of rectum to anus, various operational methods such as side-to-end coloanal anastomosis, coloplasty, colonic J-pouch, and even ileocecal pouch interposition have been used.

Considering the comparison of the primary volume to the volume after the operation, the findings of the present study revealed no significant difference between the volume increase in Group A (control) and Group B (coloplasty) (A: 20.98 vs. B: 21.75; P=0.999). Therefore, one can conclude that coloplasty operation has no superiority over coloanal anastomosis, which is a simple operation, and, consequently, it causes no more significant increase in the place of the rectum.

In Group C (J-pouch), however, a highly significant volume increase was observed compared to the control group (A: 20.98 vs. C: 118.27; P=0.029). The volume increase in Group C (J-pouch) was also significantly different from that of Group B (the coloplasty group) (P=0.030). Comparison of the pathology slides showed healing at the place of the anastomosis in all the three groups. However, the amount of inflammation in Group C (in the place of the pouch) was more in comparison to the place of the neorectum in the other two groups. 

Furthermore, coloplasty can be used as an appropriate treatment option since it is not accompanied by early dysfunction, which occurs after straight coloanal anastomosis, and long-term problems as well as the problems related to pouch evacuation, which occur after performing the colon J-pouch.

Moreover, in comparison to colon J-pouch, the chance of clinical or radiological leakage is higher in coloplasty. Therefore, the blood flow is lower at the place of the proximal anastomosis and, particularly, the anterior area in the place distal to the performance of coloplasty.^7^^,^^8^

In 1996 on Flüe et al.^8^ conducted a study and used the cecum reservoir as the neorectum by maintaining the neurovascular part of the cecum and ileum. They came to the conclusion that this method of operation was safe and practical and that it provided acceptable physiological results up to 6 months after the operation.  In a study, the chance of leakage and stricture in the CP operation was shown as 7% and 14%, respectively.^9^ In the first year after CP and colon J-pouch operations, stool fragmentation may occur, which causes the patients to defecate in 15-minute intervals. Of course, the patients may take this situation for the increase in the number of defecations by mistake.^10^ Mantyh^11^ conducted a study and revealed that the functional results were similar in both the CP group and the colon J-group.

Nowadays, after removing the rectum, colon J-pouch operation is known as the best way for connecting the colon to the anus.^7^ In comparison to straight coloanal anastomosis or CP, colon J-pouch has less chance of leakage. This is due to the better blood flow in the direction of the proximal anastomosis, which is shown through the laser Doppler technique.^12^ Colon J-pouch can increase the volume of the rectum, especially when the pouch is long; nonetheless, the increase in the length of the pouch can decrease the motility.^1^ In fact, colon J-pouch must be designed in a way that the pouch is not more than 5-6 cm because the increase in its length can lead to the problems related to evacuation.^6^

When the length of the colon J-pouch is less than 6 cm, the findings of anorectal manometry, tolerable volume and compliance, and maximum rectal volume of the initial sensation are similar in both the CP group and the J-pouch group. However, in case the length of the colon J-pouch is more than 6 cm, these parameters are better in the colon J-pouch group compared to the CP group.^13^

In spite of the fact that both CP and J-pouch have advantages as well as disadvantages and colon J-pouch is considered as the best method of operation, colon J-pouch cannot be performed on all cases. Therefore, another method of operation must be utilized in such cases. 

The results of the present study revealed that ileal J-pouch can be done in the previous location of the rectum and that it provides an appropriate volume. More studies are, however, needed to be conducted on the issue.

## Conclusion

Colon J-pouch reconstruction after rectal resection is not a suitable procedure in several cases. Therefore, this study evaluated the possibility of the creation of an ileal J-pouch interposition in an animal model and evaluated the volume of the neorectum. 

The present study is an animal study with a small sample size. If larger studies demonstrate that ileal J pouch interposition can safely create an acceptable reservoir function, this technique can be performed as a new procedure in selected cases. 

## References

[1] Koda K, Yasuda H, Suzuki M, Yamazaki M, Tezuka T, Kosugi C (2008). Reconstruction methods to achieve optimal postoperative bowel function following low anterior resection for rectal cancer. Nihon Geka Gakkai Zasshi.

[2] Derakhshani S, Hoseini SV, Asadi R, Agah S, Mohammadi-Tofigh A (2012). A new technique for pouch making to reduce the chance of leakage and low anterior resection syndrome. Journal of Medical Hypotheses and Ideas.

[3] Harris GJ, Lavery IJ, Fazio VW (2002). Reasons for failure to construct the colonic J-pouch. What can be done to improve the size of the neorectal reservoir should it occur? Dis Colon Rectum.

[4] Ho YH, Yu S, Ang ES, Seow-Choen F, Sundram F (2002). Small colonic J-pouch improves colonic retention of liquids--randomized, controlled trial with scintigraphy. Dis Colon Rectum.

[5] Hida J, Yasutomi M, Fujimoto K, Okuno K, Ieda S, Machidera N (1996). Functional outcome after low anterior resection with low anastomosis for rectal cancer using the colonic J-pouch. Prospective randomized study for determination of optimum pouch size. Dis Colon Rectum.

[6] Ho YH, Brown S, Heah SM, Tsang C, Seow-Choen F, Eu KW (2002). Comparison of J-pouch and coloplasty pouch for low rectal cancers: a randomized, controlled trial investigating functional results and comparative anastomotic leak rates. Ann Surg.

[7] Z’Graggen K, Maurer CA, Birrer S, Giachino D, Kern B, Buchler MW (2001). A new surgical concept for rectal replacement after low anterior resection: the transverse coloplasty pouch. Ann Surg.

[8] von Flüe, Degen LP, Beglinger C, Hellwig AC, Rothenbühler JM, Harder FH (1996). Ileocecal reservoir reconstruction with physiologic function after total mesorectal cancer excision. Ann Surg.

[9] Moran B, Heald R (2000). Anastomotic leakage after colorectal anastomosis. Semin Surg Oncol.

[10] Dehni N, Tiret E, Singland JD, Cunningham C, Schlegel RD, Guiguet M (1998). Long-term functional outcome after low anterior resection: comparison of low colorectal anastomosis and colonic J-pouch-anal anastomosis. Dis Colon Rectum.

[11] Mantyh CR, Hull TL, Fazio VW (2001). Coloplasty in low colorectal anastomosis: manometric and functional comparison with straight and colonic J-pouch anastomosis. Dis Colon Rectum.

[12] Hallböök O, Johansson K, Sjödahl R (1996). Laser Doppler blood flow measurement in rectal resection for carcinoma--comparison between the straight and colonic J pouch reconstruction. Br J Surg.

[13] Ortiz H, De Miguel, Armendariz P, Rodriguez J, Chocarro C (1995). Coloanal anastomosis: are functional results better with a pouch?. Dis Colon Rectum.

